# Influence of Abutment Design on Biomechanical Behavior to Support a Screw-Retained 3-Unit Fixed Partial Denture

**DOI:** 10.3390/ma15186235

**Published:** 2022-09-08

**Authors:** Guilherme da Rocha Scalzer Lopes, Jefferson David Melo de Matos, Daher Antonio Queiroz, João Paulo Mendes Tribst, Nathália de Carvalho Ramos, Mateus Garcia Rocha, Adriano Baldotto Barbosa, Marco Antonio Bottino, Alexandre Luiz Souto Borges, Renato Sussumu Nishioka

**Affiliations:** 1 Department of Biomaterials, Dental Materials and Prosthodontics, Institute of Science and Technology, São Paulo State University (Unesp), São José dos Campos 12245-000, Brazil or; 2 Center for Dental Biomaterials, Department of Restorative Dental Sciences, University of Florida (UF Health), Gainesville, FL 32611, USA; 3 Department of Restorative Dentistry & Prosthodontics, The University of Texas Health Science Center at Houston (UTHealth) School of Dentistry, Houston, TX 77054, USA; 4 Department of Oral Regenerative Medicine, Academic Centre for Dentistry Amsterdam (ACTA), The University of Amsterdam and Vrije Universiteit, 1081 LA Amsterdam, The Netherlands; 5 Department of Dentistry, Universidade São Francisco (USF), Bragança Paulista 12916-900, Brazil; 6 Midwest Dental Arts Inc., Palm Bay, FL 32909, USA

**Keywords:** finite element analysis, strain gauge, biomechanics, dental implants

## Abstract

This study aimed to evaluate the biomechanical behavior of Morse taper implants using different abutments (CMN abutment [(CMN Group] and miniconical abutments [MC Group]), indicated to support a screw-retained 3-unit fixed partial denture. For the in vitro test, polyurethane blocks were fabricated for both groups (*n* = 10) and received three implants in the “offset” configuration and their respective abutments (CMN or MC) with a 3-unit fixed partial denture. Four strain gauges were bonded to the surface of each block. For the finite element analysis, 3D models of both groups were created and exported to the analysis software to perform static structural analysis. All structures were considered homogeneous, isotropic, and elastic. The contacts were considered non-linear with a friction coefficient of 0.3 between metallic structures and considered bonded between the implant and substrate. An axial load of 300 N was applied in three points (A, B, and C) for both methods. The microstrain and the maximum principal stress were considered as analysis criteria. The obtained data were submitted to the Mann–Whitney, Kruskal–Wallis, and Dunn’s multiple comparison test (α = 5%). The results obtained by strain gauge showed no statistical difference (*p* = 0.879) between the CMN (645.3 ± 309.2 με) and MC (639.3 ± 278.8 με) and allowed the validation of computational models with a difference of 6.3% and 6.4% for the microstrains in the CMN and MC groups, respectively. Similarly, the results presented by the computational models showed no statistical difference (*p* = 0.932) for the CMN (605.1 ± 358.6 με) and MC (598.7 ± 357.9 με) groups. The study concluded that under favorable conditions the use of CMN or MP abutments to support a fixed partial denture can be indicated.

## 1. Introduction

Osseointegrated implants are used safely in the rehabilitation of partially edentulous patients; however, this treatment can present long-term complications [1]. The two main implant failures are peri-implantitis and occlusal overload. These complications can act in association or independently, and both cause marginal bone loss which can lead to implant loss in advanced cases [2,3].

In the rehabilitation of partially edentulous patients, implant placement can be affected by several factors, including bone height or the anatomy of the region [4]. The implant configuration determines the load incidence pattern in that region which consequently affect the biomechanical behavior through the stress distribution between ductile materials and microstrains at the peri-implant bone [5,6].

When an implant is under functional load, the bone tissues receive a mechanical stimulus and undergo strain. This deformation is expressed with the letter ε and is defined as the relative change in bone length, often being expressed in microstrain (με) [7]. A bone strain of 1000 means a 0.1% deformation [7], therefore bone strain in the 50–150 range allows the bone remodeling process to be kept stable, while 1500 με tends to promote lamellar remodeling to overcome the mechanical requirement. On the other hand, 3000 με is the physiological bone limit and when the load exceeds this limit, the implant may fail in this overloading scenario due to tissue damage [8].

To prevent peri-implant bone loss, it is important to know the masticatory system and the biomechanical behavior of all components of the implant rehabilitation, since the bone tissues are influenced by the occlusal loads transmitted to the implant [9]. Different implant systems provide many components/abutments, which can improve the prosthetic solution but also can hinder its correct selection. The correct abutment selection positively influences long-term treatment success, so it is essential to expand the knowledge on the subject and discuss new prosthetic possibilities [10]. In this context, the CMN abutment has a geometry with a height of 3.5 mm and an anti-rotational configuration, that is, it is indicated to support single prostheses screwed on the implant. However, the use of rotational copings on these abutments allows the manufacture of fixed partial dentures and, if their biomechanical behavior is compatible with this purpose, its indication can be extended to different configurations of prostheses on implants.

Clinical studies observed during investigations of the biomechanical behavior of implants the magnitude of the loads applied at the prosthesis/abutment level and claimed that these same stresses cannot be found at the implant-bone interface. This limitation is due to many practical and ethical obstacles to realizing a controlled clinical trial able to evaluate occlusal overloading [11].

To improve the understanding of implant rehabilitation behavior, more studies using bioengineering can be used, such as finite element analysis (FEA) and strain gauges analysis (SG) [12,13]. The numerical method using FEA allows the simulation of the load application and provides information regarding their respective stress and strain distributions [6], while the electric linear SG is a tool with high sensitivity that allows the analysis of strain peaks [14]. The association of these two methodologies allows a correct analysis of the events evaluated and aid to understand some clinical manifestations [15].

Given the foregoing, the present study aimed to evaluate the biomechanical behavior of Morse taper implants using CMN abutment (experimental group) and miniconical abutment (control group) to support a screw-retained 3-unit fixed partial denture, under axial load, using FEA and SGs. The null hypotheses were that the stress and strain distribution would be higher for the implant rehabilitation systems using CMN abutments compared with miniconical abutments.

## 2. Materials and Methods

### 2.1. Finite Element Analysis

**Figure 1:**
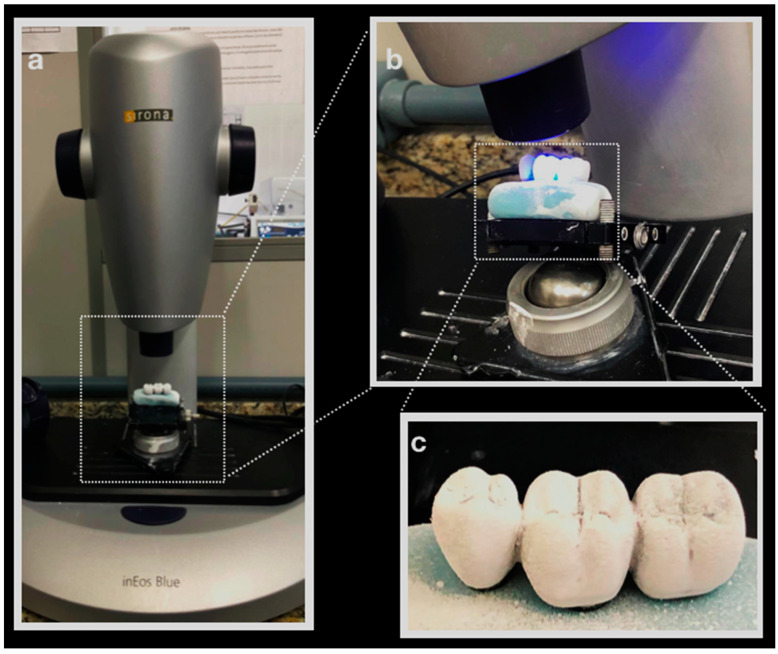
(**a**) Prosthesis on Sirona InEos Blue scanner base; (**b**) scanning; (**c**) silicone-based prosthesis with the application of Cerec Optispray (Cerec Optispray, Sirona, Bensheim, Germany).

**Figure 2:**
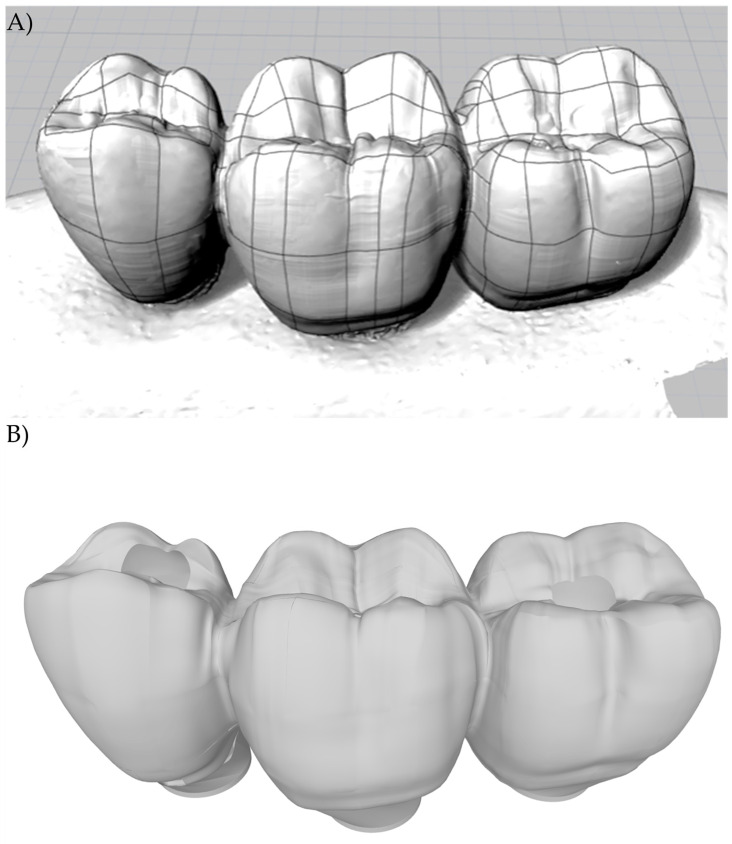
(**A**) Lines and meshes over the.STL file; (**B**) 3D model of the 3-unit fixed partial denture.

A regular Morse taper internal connection implant (4.0 × 13 mm), a CMN prosthetic abutment (Test group), a miniconical (MC) prosthetic abutment (Control Group), and a prosthetic screw (CMN: 1.8 mm; MC: 1.2 mm) were created according to the manufacturer’s dimensions (Intraoss, Sistemas de Implantes, Itaquaquecetuba, SP, Brazil) using Computer-Aided-Design (CAD) software (Rhinoceros 5.4.2, SR8, McNeel North America, Seattle, WA, USA). To simulate the 3-unit fixed partial denture, the prosthesis from the experimental model was digitized with a scanner (Sirona, InEos Blue, Beinsheim, Germany) (Figure 1) allowing the acquisition of the stereolithography (.STL) file in the dental CAD software (CEREC inLab, Sirona Dental Systems, Erlanger, Germany). Then it was exported to CAD software (Rhinoceros 5.4.2, SR8, McNeel North America, Seattle, WA, USA) (Figure 2).

To replicate the experimental model condition in FEA, two 3D polyurethane blocks were created, where three implants were positioned centrally, perpendicular to the block surface, separated on the Y axis with a distance of 3 mm between them, with the central implant separated from the X axis by 2 mm, and offset Morse taper implants were arranged.

**Figure 3:**
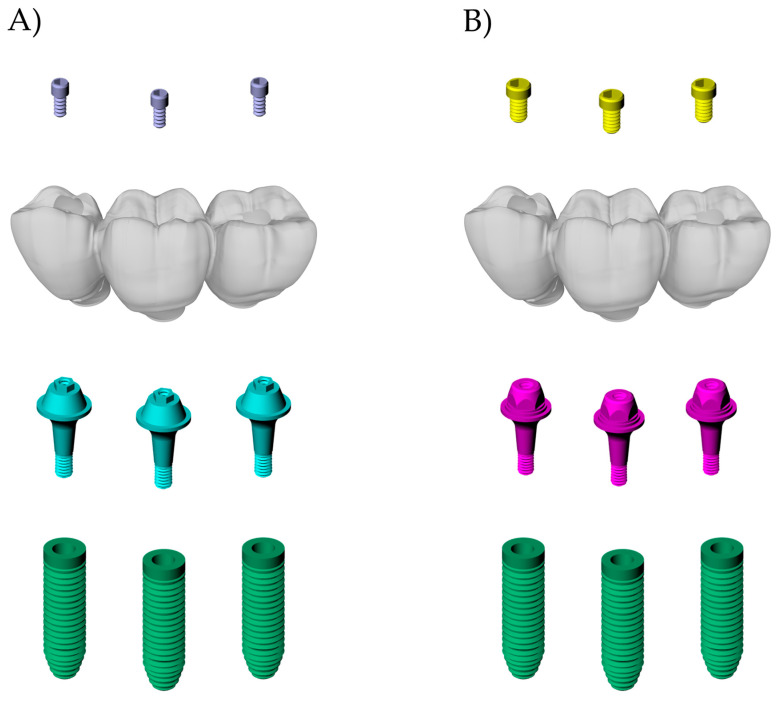
Final geometries according to the groups: (**A**) CMN; (**B**) MC.

Then, the 3-unit fixed partial dentures were positioned on their respective abutments, ensuring no misfit between them. Each abutment received its respective prosthetic screw (CMN: 1.8 mm and MC: 1.2 mm). Finally, all three-dimensional models from the CMN (Figure 3a) and MC groups (Figure 3b) were checked as volumetric solids and their geometries were saved as STEP files.

All models were imported into analysis software (ANSYS 19.2, ANSYS Inc., Houston, TX, USA) to perform static structural analysis. The material properties were assigned to each solid component as isotropic, homogeneous, and linearly elastic. The mechanical properties of polyurethane and the simulated materials were summarized in Table 1.

**Figure 4:**
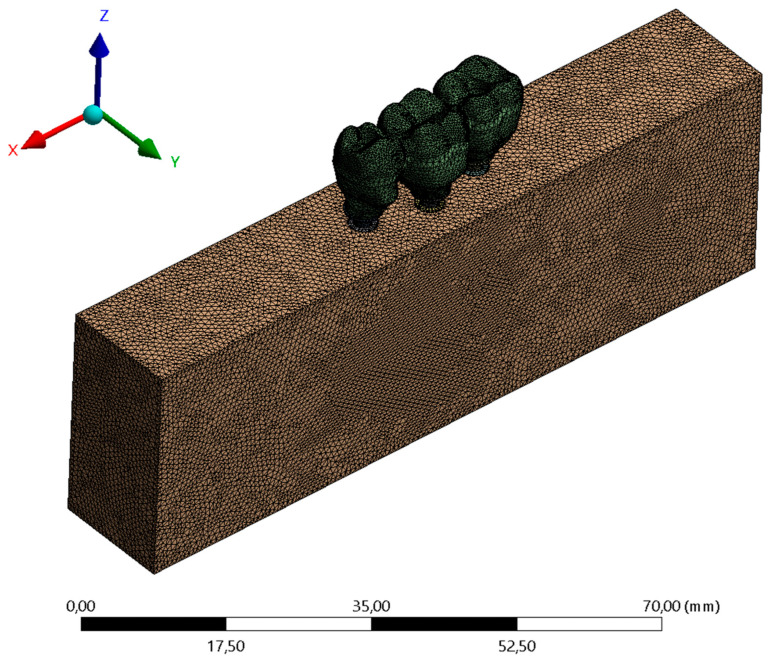
Finite element meshes.

For the implant/abutment, abutment/screw, and screw/implant contacts, the friction contact was used with a friction coefficient set at 0.3 [16], and between implant and bone, the bonded contact was used simulating complete osseointegration [6] (Figure 4).

The meshes were created automatically by selecting the 0.3 mm parameter of tetrahedral elements. Then, a 10% convergence test [6,12,13] determined 390.548 elements and 702.122 nodes for the CMN group and 411.192 elements and 690.548 nodes for the MC group. For each axial load: point A (center of implant retention screw #13); point B (center of implant retention screw #14), and point C (center of implant retention #15) an analysis configuration was inserted, and the load was defined as vectorial in the Z axis with 300 N (30.6 Kgf). The fixation was defined on the bottom surface of the polyurethane block (Figure 4).

Among the many possibilities for the analyses of the results, maximum principal stress was selected to evaluate ductile materials and the microstrains solutions were selected to evaluate the substrate. Both methodologies are widely known within the engineering field and are most referenced in studies of the stress behavior of metals and polyurethane/bone [6,12,13].

### 2.2. Strain Gauge Analysis

For the strain gauge analysis, 20 polyurethane blocks (95 × 45 × 30 mm) (Polyurethane F16 Axson, Cercy, France) were manufactured to simulate an isotropic substrate for each group (N = 20, *n* = 10). To prevent pores, the polyurethane resin polymerization was carried out in a vacuum pressurizer (Protecni, Araraquara, São Paulo, Brazil). After the polymerization, the blocks were removed from the matrix and their surfaces were polished with progressive sandpaper (#220 to #600 grit) underwater.

A metallic die [17] was used to standardize the three implant placements (4.0 × 13 mm, Intraoss, Sistemas de Implantes, Itaquaquecetuba, SP, Brazil), perpendicular to the surface, at the bone level, and in the “offset” configuration. The respective abutments were installed with the aid of a manual torque wrench and the manufacturer’s guidance, 32 N.cm for the CMN and 20 N.cm for the miniconical abutments (Intraoss, Sistemas de Implantes, Itaquaquecetuba, SP, Brazil). Rotational plastic copings developed for the study were screwed onto the CMN abutments and conventional rotational plastic copings were screwed onto the miniconical abutments (Intraoss, Sistemas de Implantes, Itaquaquecetuba, SP, Brazil).

After the waxed first prosthesis, a condensation silicone template (Speedex, Coltene, Altstätten, Switzerland) was fabricated to ensure that the anatomy of the 3-unit fixed partial denture can be replicated for the other specimens. The occlusal part of the template was removed, and the occlusal part of the prosthesis was copied again with type IV gypsum (Durone, Dentsply Ind. e Com. Ltd. a Petrópolis, Brazil). A total of ten waxed 3-unit fixed partial dentures were completely cast in Ni-Cr alloy (Wironia Light Bego, Bremen, Germany) by lost wax technique for each group. After casting, the specimens were bench-cooled and devested by airborne particle abrasion with 50 μm aluminum oxide. Finally, finishing and polishing were performed. All prostheses were screwed on the abutments according to the manufacturer’s recommendation by using a torque wrench (Intraoss, Sistemas de Implantes, Itaquaquecetuba, SP, Brazil).

**Figure 5:**
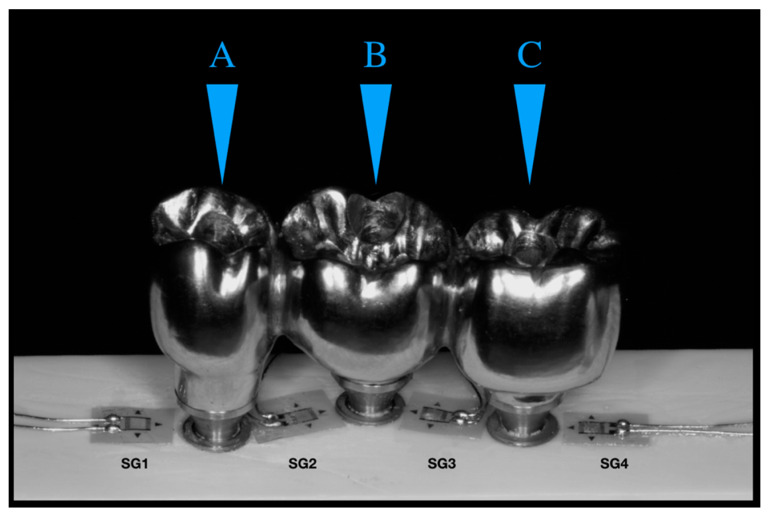
Strain gauges are arranged between the implants and the application load points. Letters showing the different loading points.

The surface of the 20 blocks was cleaned with isopropyl alcohol and four unidirectional linear SGs model PA-06-060BA-120-L (Excel Sensores Ind. Com. Exp. Ltd. a, Taboão da Serra, São Paulo, Brazil, resistance 120 Ω; gauge length: 1.5 × 1.3 mm) was bonded to the surface of each block with cyanoacrylate adhesive (Super Bonder Loctite, São Paulo, Brazil). SG1 was placed mesially adjacent to implant #13, SG2 and SG3 were placed mesially and distally adjacent to implant #14, and SG4 was placed distally adjacent to implant #15 [14] (Figure 5).

Each strain gauge outlet was measured using a multimeter (Minida ET 2055: Minida São Paulo, Brazil), ensuring that the connector output had the same resistance (120 Ω) [18]. Four electrical cables were installed at the outputs and connected to an electrical signal conditioning apparatus (Model 5100B Scanner–System 5000–Instruments Division Measurements Group, Inc. Raleigh, Carolina do Norte–USA, FAPESP proc: 07/53293-4) to record variations in electrical resistance and convert them in microstrain (με/με).

A 2 mm diameter rounded tip present in the load application device [16] was used, which allowed constant axial loads of 30.6 Kgf. Loads were applied at 3 axial points: point A (center of implant retention screw #13); point B (center of implant retention screw #14), and point C (center of implant retention #15). The microstrains generated by load application at the 3 axial points were recorded by the four strain gauges. The same procedure was performed for each group (*n* = 10), repeating three loads per application point.

The obtained data were submitted for exploratory analysis of normality (Shapiro–Wilk). The SGs and FEA data did not attend the normality (*p* < 0.05) and were submitted to Mann–Whitney and Kruskal–Wallis followed by Dunn’s multiple comparison test, with a significance level of 5% (R-project software, version 3.2.0, 2016).

## 3. Results

The results obtained from SGs were regarded as the analysis of peri-implant microstrains for the CMN and MC groups (Table 2).

The strain peaks were 1506 and 1390 με for the CMN and MC groups, respectively. The Mann–Whitney test demonstrated that “Group” were no statistical difference for microstrains values (W = 7282; *p* = 0.879) between CMN (645.3 ± 309.2 με) and MC (639.3 ± 278.8 με) groups. The Kruskal–Wallis test, followed by the Dunn’s multiple comparison test (α = 5%) demonstrated that the load application points were not statistically significant (df = 2; x2 = 3.22; *p* = 0.199) for microstrains values in the points A, B, and C: 611.3 ± 281.9; 661.6 ± 234.7, and 654.0 ± 353.6 με, respectively.

**Figure 6:**
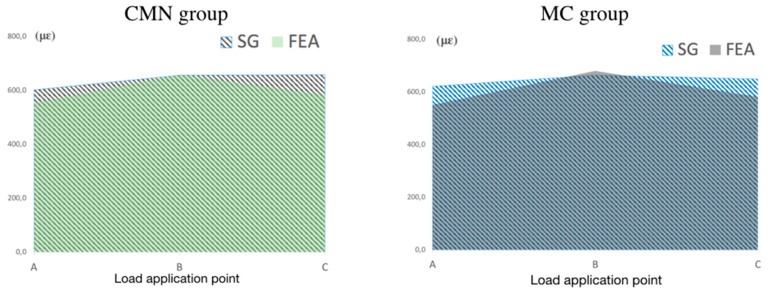
Microstrains mean the CMN and MC groups for both methodologies according to the different loading points.

For the validation of the theoretical models (FEA), the microstrain values were calculated in the same positions and directions as the experimental models (SG). Peri-implant microstrain was adopted as an analysis criterion for the correlation between methodologies, allowing its validation. To each loading application point (A, B, and C), the values of the four SG positions were measured, and the means were calculated, plotted on graphs, and overlapped to show the compatibility in the results of the theoretical and experimental models from CMN and MC groups. To the CMN group, the microstrain means for the used methodologies (FEA: 605.1 με; SGs: 645.3 με) presented a difference of 6.33%, and to the MC groups (FEA: 598.8 με; SGs: 639.3 με) the difference was 6.4% (Figure 6).

Regarding the FEA, quantitative and qualitative results were obtained, since this analysis showed that CMN and MC groups showed much approximation in their strain peaks, 1189 με and 1192 με, respectively. Corroborating with the results obtained in the experimental models (SG), the strain values observed in the theoretical models (FEA) were also presented within the physiological limits. According to the Mann–Whitney test, it was not possible to observe a statistical difference (W = 0.88; *p* = 0.932) between CMN (605.1 ± 358.6 με) and MC (598.7 ± 357.9 με) groups. Regarding the “load application point” factor, the following microstrains (με) means were observed for points A, B, and C: 553.2 ± 421.5; 670.3 ± 56.8, and 582.2 ± 462.5 με, respectively. The Kruskal–Wallis followed by Dunn’s multiple comparison test (α = 5%) demonstrated there was no statistical difference for this factor (df = 2; x2 = 0.08; *p* = 0.960).

Evaluating the stress peaks in the structures of each group, it was possible to observe that the highest stresses were concentrated in the abutments, with a value of 98.5 MPa for the MC group and 91.6 for the CMN group. Then, the stresses peaks showed lower values in the implants (MC: 57.8 MPa; CMN: 56.5 MPa) and even lower in the prostheses (MC: 12.1 MPa; CMN: 13.4 MPa) and prosthetic screws (MC: 13.2 MPa; CMN: 13.4 MPa) (Table 3).

**Figure 7:**
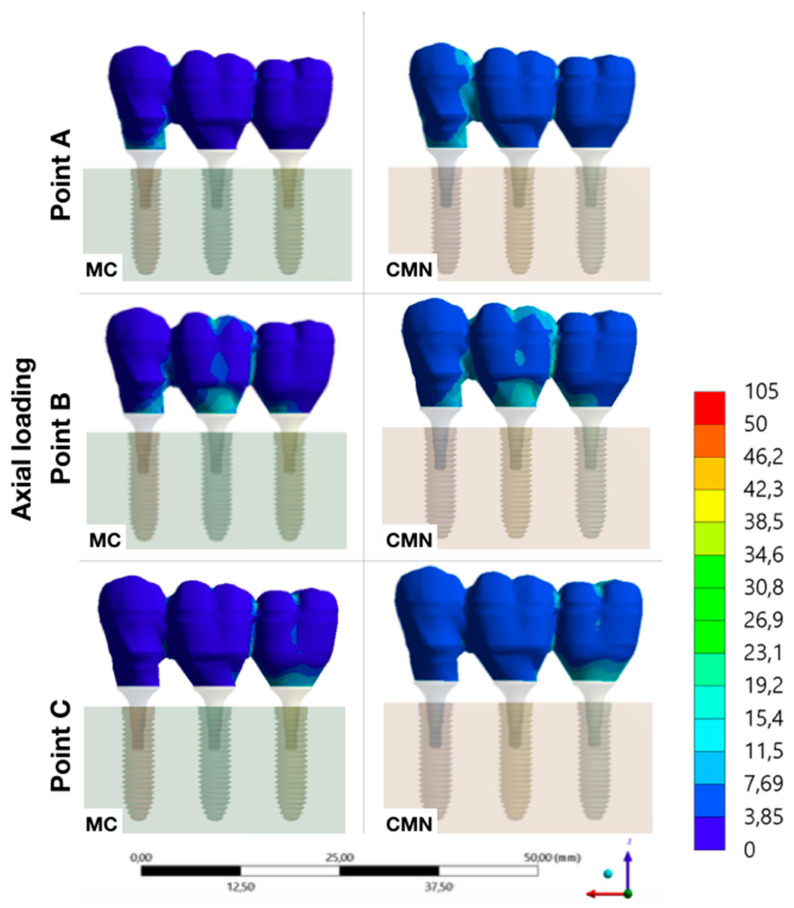
Maximum principal stress (MPa) at the prosthesis for the load application at points A, B, and C (CMN and MC groups).

**Figure 8:**
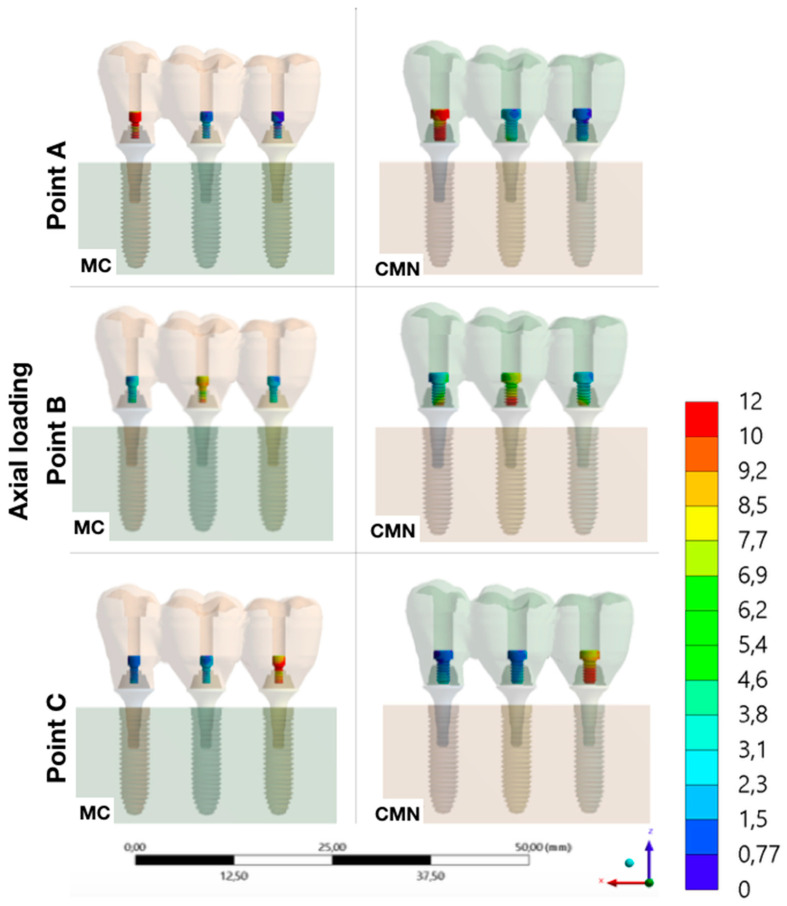
Maximum principal stress (MPa) at the screws for the load application at points A, B, and C (CMN and MC groups).

**Figure 9:**
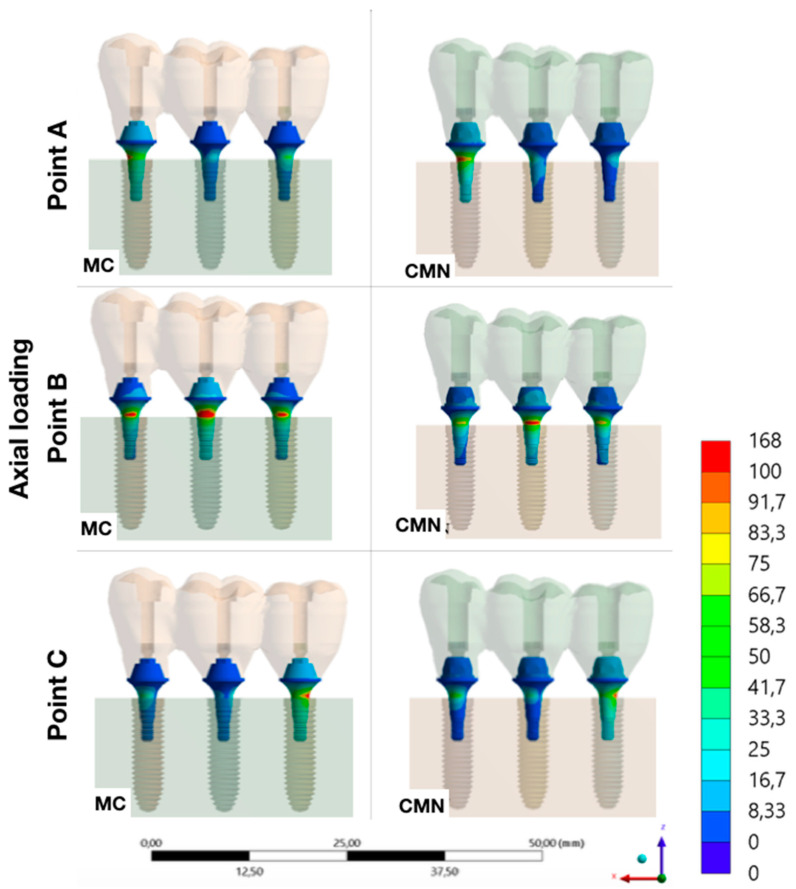
Maximum principal stress (MPa) at the abutments for the load application at points A, B, and C (CMN and MC groups).

**Figure 10:**
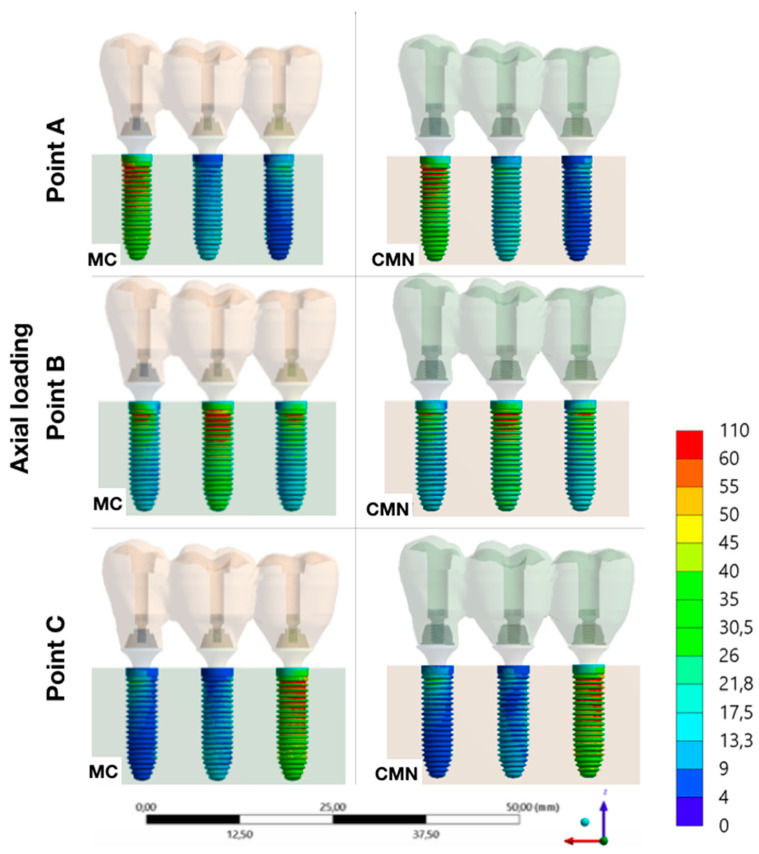
Maximum principal stress (MPa) at the implants for the load application at points A, B, and C (CMN and MC groups).

**Figure 11:**
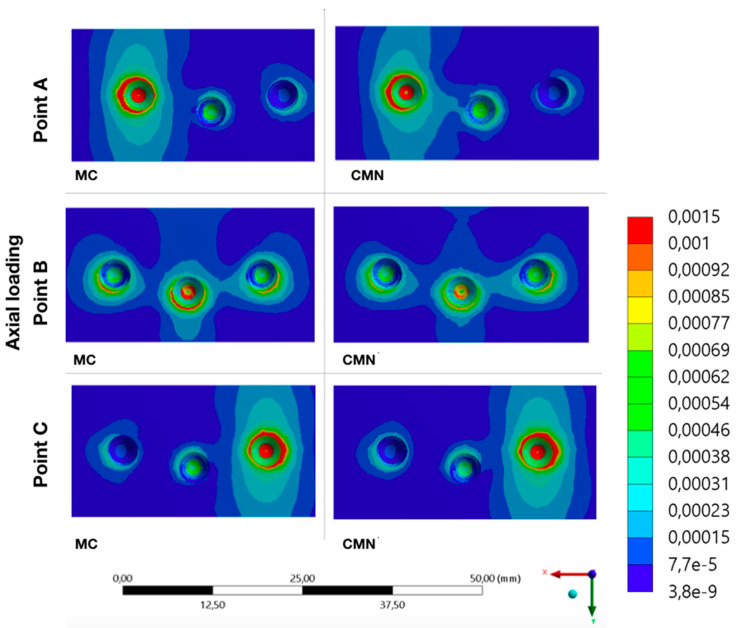
Microstrains (με) at the substrate for the load application at points A, B, and C (CMN and MC groups).

It was possible to observe the biomechanical behavior of each group with different load application points, adopting the maximum principal stress as stress criteria for ductile materials and the microstrains for polyurethane (Figure 7, Figure 8, Figure 9, Figure 10 and Figure 11).

## 4. Discussion

The wide use of computational methodologies (FEA) to evaluate the biomechanical behavior of implants is due to their high efficiency and low investment for their implementation since clinical or laboratory methodologies have limited use by non-destructive means [17,18,19]. To evaluate the accuracy of this method or to validate these theoretical models, the compatibility of its results with laboratory experiments is mandatory [6]. The use of SGs to validate computational models is based on the technology used in its small diameter devices that present a high precision to measure the strain of surfaces [13,20]. In this context, the present study used SGs to validate the FEA models, allowing the analysis of the biomechanical behavior of all structures of the rehabilitation systems, that is, investigations that are difficult to access by laboratory methodologies.

Some authors have used the findings of their laboratory tests using polyurethane and correlated with their three-dimensional models using cortical and cancellous bone in their simulations, which led to a discrepancy in the obtained data by the two methodologies [21]. Thus, corroborating with other studies [22,23], the present study used polyurethane for bone simulation in the laboratory and theoretical models, since it is an isotropic material validated in the literature for these simulations, as it presents Young’s modulus between the cortical and cancellous bone. The use of polyurethane facilitates the standardization of experiments and ensures greater compatibility between the results of SGs and FEA.

The main methodologies to evaluate stress distribution in laboratory models are photoelasticity and strain gauges. Among them, SGs have been widely used because it allows measurements of the surface strain of a given material under static loading [19,24]. This high precision to measure the surface behavior of solids, makes strain gauge a very effective methodology in the investigation of the biomechanical behavior of implant-supported rehabilitation since in the case of two materials with different mechanical properties (implant × bone) when the implant is loaded, these stresses are transmitted in the region of its first contact, that is, on the surface of the surrounding bone [25]. Other studies that used SG to investigate the behavior of implant-supported prostheses showed a large standard deviation, which can be attributed to the complexity of the restorative system when using completely cast or overcast abutments [14,18]. Similarly, the present study also showed a large standard deviation for the two groups evaluated using completely cast abutments.

When there is a discrepancy between the results presented by two methodologies, the lack of compatibility between them and the existence of a theoretical model that is not validated or inaccurate is evident [6]. Considering that these models are validated when the results of both methodologies are similar, the present study considered microstrain as a stress criterion to validate the theoretical model, with a difference of 6.3% in the CMN and 6.4% in the MC group among adopted methodologies, the theoretical models were validated.

The null hypotheses of the study were rejected since the use of CMN abutments to support a 3-unit fixed partial denture did not show any difference in the biomechanical behavior in comparison with the use of MC abutments. The increased height of 3.5 mm and the presence of an anti-rotational geometry of the CMN abutment suggest a higher concentration of stress (>10%) and even greater bone strains, once these components in multiple prostheses promote pre-load stress caused by the misfit or by the disrupted of prosthesis passivity [26]. This same abutment was already investigated, using the finite element method, supporting multiple prostheses with two and three implants; however, the authors evaluated only the stress distribution in the ductile materials of the rehabilitation systems [5], without contemplating the prosthetic screw behavior, that is considered to be the most fragile component of implant-supported rehabilitation [1,27]. Therefore, the present study extrapolated the presented results above and evaluated the influence of these abutments on bone strains with different methodologies, and evaluated the biomechanical behavior of all structures using validated FEA models. All analyses of the CMN abutment were related to miniconical abutments (control), that is, exclusive components of multiple prostheses since their geometry only allows the stability of the prosthesis when it is joined to other implants [28]. According to others [21], greater relevance can be considered in the presented data, since the association of two or more methodologies allows a greater understanding of clinical behavior.

More important than assessing loading is to investigate its influence on bone tissues [29]; therefore, it is essential to investigate and understand the biomechanical behavior of the components and their influence on bone strains, as these deformations may be beyond the physiological limit (>3000 με) without any damage to the components of the implant system [8,30]. In this context, axial loading of 300 N was applied to the center of the three retention screws as it is the average found in the posterior region [31] and it was possible to observe that besides presenting great similarity in the biomechanical behavior, the evaluated groups did not present pathological microstrains for any loading points. Some factors may have contributed to this low strain observed, for example, the parallelism between the implants, the “offset” configuration, axial loading, and even the presence of a platform switching connection [32].

It was also possible to observe that axial loads on the central implant (point B) presented lower strains with more homogeneous distribution. However, the bone strain exhibited a behavior pattern when the loading was applied in more peripheral regions (points A and C), the result of the originated stresses was a fulcrum in the implant closest to where the load was applied and a rotation tendency of the prosthesis, which generated higher bone strains. Other authors who also investigated the biomechanics of 3-unit fixed partial dentures on implants have already reported this same behavior [6,33].

The performance of rehabilitation with implants can be determined by comparing the stress peaks in the investigated components [34] and, according to the biomechanical behavior of ductile materials, a higher stress concentration was observed (maximum principal stress) in the abutments, regardless of the evaluated group. The titanium used in implantology has a mechanical strength of approximately 2000 MPa [35], therefore it is evident that there is a possibility of the dental implant reaching a pathological bone strain before the failure of any component of the rehabilitation system. Corroborating with others [5], who evaluated the same geometry as the CMN abutments, it was possible to observe that the stresses were also concentrated in the region of first contact between the abutments/implants, which should be evaluated with caution since there is a greater mechanical requirement in the region of abutment constriction. Other authors also agree that the abutment design may be the most critical of all the ductile structures; however, as the retention screw is exposed to proportional stresses to the abutment, the damage probability is greater in the screw [34].

Under static axial loads, the stresses are homogeneous distribution over the structures of the implant-supported rehabilitation [36] since this load limits the horizontal displacements of the prosthesis about its initial positioning with its retention screws [37]. The present study evaluated abutments that require prosthetic screws with different diameters and, given the efforts required in the simulations, it was possible to observe a good performance for screws of 1.2 (MC abutment) and 1.8 mm (CMN abutment). Other authors have observed that non-axial or oblique loadings concentrate greater stresses on the prosthetic screw [27,38,39]. Any restoration in the oral cavity under multiaxial and/or oblique loading and loosening or fracture of the screw is one of the main mechanical complications of implant restorations.

Regarding implant behavior, the two groups evaluated in the present study presented similar data. Other authors who evaluated implants with Morse tapper conical connections also observed that these stresses tend to be located on the external part of the implant, close to the first threads [5,6]. Considering that several implant systems offer reduced implant diameters for the same abutment, there is invariably thinness in areas with high mechanical demands, which can lead to fracture of the implants in the cervical region and peri-implant bone loss due to pathological microstrains [37,40].

The 3-unit fixed partial prosthesis was simplified and fabricated in Ni-Cr for the two groups evaluated. Such material is not applicable in clinical practice for the manufacture of prostheses on implants. However, in previous studies, authors have observed that Young’s modulus and the biomechanical behavior of the prostheses can be proportional since the same geometry is maintained [38,41,42,43,44]. Therefore, the simplification of the prostheses does not seem to influence the biomechanical behavior of the models, allowing an effective analysis of the abutment of interest. The stresses were located predominantly in the region where the prosthesis was seated and in the regions of the connectors closest to where the load was applied, corroborating with data from other authors [5,39,45].

There are some limitations in this computational simulation and experimental study: Factors inherent to the complexity existing in the oral cavity were not summed, such as the variation in humidity, temperature, and pH. The use of homogeneous structures in 3D models, which do not allow internal defects in their geometries, is also a limitation. However, these limitations do not invalidate the results exposed in the present study but suggest caution in their interpretation and the need to associate the data exposed with others available in the literature. Thus, the need for further studies using non-axial loads, simulations with implants outside the ideal position, as well as long-term clinical studies for a better understanding of the behavior of these CMN abutments supporting multiple prostheses is evident.

## 5. Conclusions

Therefore, after model validation, it can be concluded from this study that, under favorable conditions, the prosthetic rehabilitation with CMN abutments showed similar biomechanical behavior with the MC abutments, without leading to a deleterious bone strain. Regardless of the abutment type, the highest stress concentration occurred in the contact region between the abutment and implant.

## Figures and Tables

**Table 1:** Properties of the material used in the study.

Material	Young Modulus (GPa)	Poisson Ratio	Reference
Titanium	110	0.32	[12]
Nickel Chromium	206	0.30	[6]
Polyurethane	3.6	0.30	[12]

**Table 2:** Microstrains (με/με) and standard deviation (SD) according to the load application point.

		SG1 (SD)	SG2 (SD)	SG3 (SD)	SG4 (SD)
	Point A	847.9 (251.9)	808.3 (183.1)	433.6 (106.3)	394.7 (92.3)
CMN	Point B	840.1 (147.4)	571.0 (154.0)	454.1 (246.9)	797.9 (259.9)
	Point C	436.3 (208.1)	632.9 (486.5)	505.2 (290.5)	1022.5 (265.5)
	Point A	565.4 (310.7)	905.6 (312.5)	516.6 (196.1)	407.3 (106.1)
MC	Point B	601.4 (198.3)	684 (142.8)	676.3 (222.3)	706.5 (252.2)
	Point C	1073.6 (233.7)	473.2 (151.0)	445.1 (212.7)	664.7 (227.1)

**Table 3:** Maximum principal stress peaks (MPa) in the structures of the MC and CMN groups.

Structures	Group	Stress Peak (MPa)
Prosthetic screw	CMN	13.4
	MC	13.2
Prosthesis	CMN	13.4
	MC	12.1
Dental implant	CMN	56.5
	MC	57.8
Abutment	CMN	91.6
	MC	98.5

## Data Availability

Data are available upon request.
